# Development and Validation of a 10-Year-Old Child Ligamentous Cervical Spine Finite Element Model

**DOI:** 10.1007/s10439-013-0858-7

**Published:** 2013-07-02

**Authors:** Liqiang Dong, Guangyao Li, Haojie Mao, Stanley Marek, King H. Yang

**Affiliations:** 1 The State Key Laboratory of Advanced Design and Manufacturing for Vehicle Body, Hunan University, Changsha, Hunan China; 2 Bioengineering Center, Wayne State University, Detroit, MI USA

**Keywords:** Pediatric cervical spine, Finite element method, Tension fracture, Flexion/extension, Growth plate

## Abstract

Although a number of finite element (FE) adult cervical spine models have been developed to understand the injury mechanisms of the neck in automotive related crash scenarios, there have been fewer efforts to develop a child neck model. In this study, a 10-year-old ligamentous cervical spine FE model was developed for application in the improvement of pediatric safety related to motor vehicle crashes. The model geometry was obtained from medical scans and meshed using a multi-block approach. Appropriate properties based on review of literature in conjunction with scaling were assigned to different parts of the model. Child tensile force–deformation data in three segments, Occipital-C2 (C0–C2), C4–C5 and C6–C7, were used to validate the cervical spine model and predict failure forces and displacements. Design of computer experiments was performed to determine failure properties for intervertebral discs and ligaments needed to set up the FE model. The model-predicted ultimate displacements and forces were within the experimental range. The cervical spine FE model was validated in flexion and extension against the child experimental data in three segments, C0–C2, C4–C5 and C6–C7. Other model predictions were found to be consistent with the experimental responses scaled from adult data. The whole cervical spine model was also validated in tension, flexion and extension against the child experimental data. This study provided methods for developing a child ligamentous cervical spine FE model and to predict soft tissue failures in tension.

## Introduction

Spinal injuries in children have a higher morbidity and mortality rate compared with those of adults.^11^ About 75% of pediatric spinal injuries are in the cervical region compared to 14% in the thoracic and 11% in the lumbar regions.^4^ Motor vehicle crashes account for the majority of injuries in the pediatric population.^4^ Cervical spine injuries in children are different from those in adult, due to different anatomical and physiological features.^12^ These differences include the relatively large head mass,^22^ large ligamentous laxity,^30^–^32^ shallow angulations of facet joints,^25^ developing ossification of vertebrae,^14^ and immature neck musculature.^34^ Additionally, the pediatric spine has unique anatomical features like growth plate and apophyseal ring_ENREF_1.

Animal tests have been used to obtain pediatric cervical spine responses in tension and compression.^13^,^44^ Cadaveric functional spinal unit tests^31^ and intact cervical spinal osteoligamentous tests^40^ were also performed to identify tensile properties of human pediatric subjects. However, these tests only quantify overall biomechanical responses; internal responses such as strain and stress cannot be calculated directly due to irregular shaped vertebrae. These internal responses are important to predict injury locations and mechanisms.^62^ Finite element (FE) models can be used to calculate these responses.

A number of adult cervical spine FE models have been developed.^24^,^42^,^57^,^65^ There were few pediatric cervical spine FE models reported, partially due to a lack of test data for model validation. The first pediatric human cervical spine FE model was developed by Kumaresan *et al*.^29^ Three C4–C6 segment models (representing one, three, and 6 years old) were developed by scaling down an adult model 33 years of age,^28^ and then adjusted for pediatric facet angles and the size of the nucleus. Effects of ossification and geometric changes were calculated by comparing flexibilities predicted by the adult and pediatric models. The material properties of annulus fibers for the three child neck segment models were scaled from the adult, but all other respective anatomical structures for the three child models were assumed to have the same material properties without distinction of age differences. Mizuno *et al*.^37^ developed a 3-year-old total human FE model scaled from an adult model (THUMS AM50, Toyota, Japan). The basic anthropometry data was based on the dimensions of a Hybrid III dummy. The elastic modulus scale factor of the bone was based on Irwin and Mertz.^22^ The failure stress and strain scale factors of bone were based on research reported by Currey *et al*.^7^ Material properties used to simulate soft tissues were the same as adult models adopted by THUMS. The model was validated based on 3-year-old dummy tests for the spine flexion and chest compression, while validations of other body regions were not reported. The aforementioned child cervical spine FE models were scaled from the adult, which neglected the different anatomical features uniquely presented in a child.

Meyer *et al*.^36^ developed a 3-year-old child cervical spine model based on detailed human child geometry. The vertebral bodies were assumed to be rigid and the intervertebral discs were modeled as elastic material without partition of the annulus and nucleus. Adult material properties were assumed for ligaments in their child model. The scale factors selected for the mass and moment of inertia of vertebrae and head were based on Irwin and Mertz.^22^ This model was not validated against pediatric human experimental data.

When the first generation airbag was implemented in vehicles to provide passive protection, some airbags were too forceful and resulted in deaths to small female and child occupants in frontal crash. As a result, government regulations demand deactivation of the passenger airbag for under 6-year-old child seating in the front passenger seat. The 10-year-old occupant is situated between the 6-year-old child group and adult small female and exposure to an inflating airbag is possible. Since an airbag generates tensile loading to the neck, a 10-year-old child model validated against tensile biomechanical data would be useful in the design and evaluation of age-appropriate parameters for airbag deployment.

Some studies^20^,^23^,^53^ indicate that child pedestrians 5–12 years of age are at the highest risk of being injured by a vehicle. For child pedestrians 8–12 years of age, the rate of injury per kilometer or time spent on the road, or per road crossing, was the highest compared to 3–7 YO and 13–17 YO groups.^23^ Also, it has been suggested that older (8–12 years old) children using seat belts don’t derive similar protection in crashes as younger (under 8) children using child restraints.^17^ These injury statistics point towards the need to have a 10-year-old biofidelic child model to study a variety of crash scenarios to derive countermeasures. In order to overcome the deficiencies in existing child cervical spine models and to improve the safety of 10-year-old child in car crashes, the aims of this study were to develop a 10-year-old ligamentous cervical spine FE model based on detailed geometry of pediatric subject and investigate the tissue-level failure under tension.

## Materials and Methods

CT scans of one subject (9.8 years old) at 5 mm slice thickness and a screen resolution of 1024 pixels were used to segment the bony sections of the model. This subject was selected because his neck length and circumference were close to the average anthropometry of 10-year-old child.^49^,^54^ Geometries of ligaments, cartilages, and intervertebral discs not visible in the CT images were filled in between bony segments based on adult anatomy^1^,^38^ and child spinal models.^29^,^36^,^46^ A multi-block approach was adopted in this study to generate vertebral body meshes efficiently (ANSYS ICEM CFD/HEXA 12.0, Ansys, Canonsburg, PA, USA). Hypermesh 10.0 (Altair, Troy, MI) was used for generation of the remaining meshes. The coordinate system of the entire model was defined with the positive *x*-axis pointing to the anterior direction, *y*-axis pointing to the left, and *z*-axis pointing to the superior direction.

### Material Modeling

**Figure 1:**
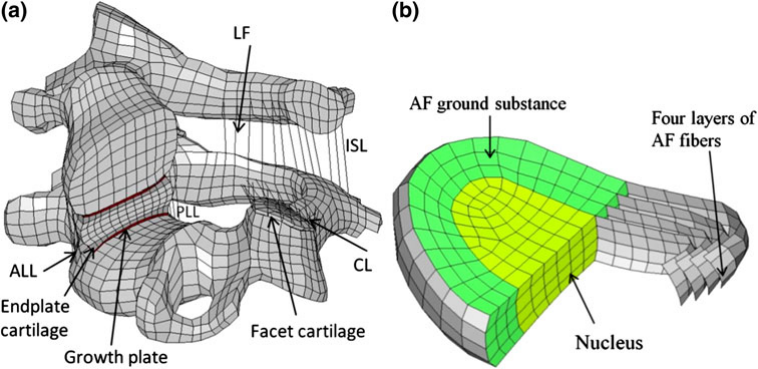
(a) Sectioned isometric view of the C4–C5 segment model (ALL—anterior longitudinal ligament; PLL—posterior longitudinal ligament; CL—capsular ligament; LF—ligamentum flavum; ISL—interspinous ligament) and (b) the three components used to represent the intervertebral disc

Material laws and properties assumed for the 10-year-old child cervical spine model are summarized in Table 1. The cancellous bone and cortical bone were modeled as isotropic elastic–plastic material (*MAT_POWER_LAW_PLASTICITY in LS-DYNA). Based on quantitative CT densities reported for child and adult vertebral cancellous bone,^18^ the scale factor used to scale the adult material parameters of cortical and cancellous bone was calculated to be 0.805. The material properties of the endplates were defined as one-third of those for cortical bones, as assumed by Panzer and Cronin.,^42^ which was also supported by a figure reported in Denoziere and Ku^8^ based on experimental data. The growth plate was modeled as cartilaginous tissue between the vertebral body and endplate cartilage^46^ (Fig. 1a).

**Table 1:** Material properties assumed for the 10-year-old cervical spine FE model and the scale factors used to determine properties of the child

Name	Element type	Material model	Material parameters	Scale factors [ref]	Properties references
Cortical bone	Hexahedral	Isotropic elastic–plastic	*E* = 13.44 GPa, γ = 0.3	0.805^b^	6
			*k* = 355 MPa, *N* = 0.277		
Cancellous bone	Quadrilateral	Isotropic elastic–plastic	*E* = 241 MPa, γ = 0.3	0.805^b^	27
			*k* = 5.73 MPa, *N* = 0.274		
Endplate	Quadrilateral	Isotropic elastic–plastic	*E* = 4.48 GPa, γ = 0.3		
			*k* = 118 MPa, *N* = 0.277		
Growth plate	Hexahedral	Isotropic elastic	*E* = 25 MPa, γ = 0.4		5,15,55
Endplate cartilage	Hexahedral	Isotropic elastic	*E* = 23.8 MPa, γ = 0.4	^a^	56
Annulus ground substance	Hexahedral	Hill foam	*n* = 2	0.782^63^ ^ b^	16,21,51
			*C*_1_ = 0.090 MPa, *b*_1_ = 4		
			*C*_2_ = 1.643 MPa, *b*_2_ = −1		
			*C*_3_ = −0.699 MPa, *b*_3_ = −2		
Annulus fibers	Quadrilateral	Orthotropic elastic	Stress-stretch curve	0.782^63^ ^ b^	19
Nucleus	Hexahedral	Fluid	*K* = 1.72 GPa	^a^	57
Facet cartilage	Hexahedral	Isotropic elastic	*E* = 10 MPa, γ = 0.4	^a^	56
Ligaments	Bar	Non-linear		0.893^63^ ^ b^	3,61,63
Dimensional scale factor *G*_S_				0.723^35^	

*E*, Young’s modulus; *γ*, Poisson’s ratio; *k*, strength coefficient; *N*, hardening exponent; *n*, *C*_*i*_, *b*_*i*_, material constant; *K*, Bulk Modulus

^a^Indicates the material parameters was the same as the values of adult

^b^The scale factors were used to scale adult material parameters to child ones

The cancellous bone was modeled using hexahedral elements. Cortical bone and endplates were modeled using shell elements. The thicknesses of the cortical bone and endplate for adult range from 0.41 to 0.70 mm with an average of 0.5 and 0.6 mm for cortical bone and endplate, respectively.^41^ Because these structures were too thin to be detected accurately from medical images, it has been common practice to assign uniform thicknesses for adult FE neck models.^42^,^65^ For the 10-year-old child model, a uniform thickness of 0.37 and 0.45 mm, respectively, was assumed for the cortical bone and endplate. These values were based on the dimensional scale factor *G*_S_ (Table 1) reported by Mertz *et al*.^35^

The intervertebral disc was modeled into three parts as shown in Fig. 1b. The ground substance of the annulus fibrosus (AF) was divided into three layers in the radial direction and the AF fiber laminae was represented by four layers of membrane elements. The volume of the fiber lamellae was approximately 20% of the total annulus volume.^47^ The cross-sectional area of the nucleus was about 50% of the total cross-sectional area of the disc.^45^

The AF fiber lamellae consist of collagen fibers oriented at about 25 or 155° on adjacent layers near the outer region and at about 45 or 135° for the inner region.^2^,^51^ This complex structure was simplified into four layers (Fig. 1b) with the fiber orientations in the outer most (fourth) layer defined as 25 and 155°, third layer defined as 32 and 148°, second layer defined as 39 and 141°, and the innermost (first) layer defined as 45 and 135°, respectively. The only available mechanical test data for single lamella was from the lumber region of adult specimens reported by Holzapfel *et al*.^19^ The current study used the same method adopted by Panzer and Cronin^42^ to interpolate the stress-stretch curves for the four simplified layers. The scale factor reported by Yoganandan *et al*.^63^ (Table 1) was then used to scale these curves to represent the properties of 10-year-old lamellae.

The Hill foam material model available in LS-DYNA was used to model the AF ground substance. Parameters (Table 1) needed for the material model were determined based on experimental data obtained in the uniaxial tension,^16^ confined compression,^21^ and unconfined compression^51^ tests on samples aligned in the radial direction. This is the same method used by Panzer and Cronin^42^ to identify the material properties of the ground substance. The nucleus pulposus was modeled using fluid elements with a bulk modulus of 1.72 GPa.^58^

To the best of the authors’ knowledge, few studies have been conducted to simulate the intervertebral disc failure in an FE model. One method was reported by DeWit and Cronin^9^ for an adult C4–C5 segment. The authors used a tie-break contact between the endplate cartilage and AF with a pre-defined critical stress to mimic failure. The reason for this was that the majority of failure in tensile testing of bone-disc-bone specimens occurred at the endplate cartilage-AF boundary.^26^ DeWit and Cronin calculated the failure tensile force for the intervertebral disc based on the average failure tensile stress reported by Kasra *et al*.^26^ The critical stress was then calculated by applying this failure tensile force to cross-sectional layers of AF fibers. In the current study, the element deletion method was used to simulate failures of the growth plate and endplate cartilage when element stress exceeded the pre-defined critical stress. The The DeWit and Cronin’s method was used to calculate the failure stresses as shown in Table 2. The failure stress of the growth plate and endplate cartilage was set at the same value. In order to calculate the failure stresses for child, the following formulation was used to obtain the failure tensile forces for child based on adult data:

**Table 2:** Failure stress calculated for C4–C5 and C6–C7 intervertebral discs based on tensile failure forces of intervertebral discs

	Failure tensile force for adult (N)	Scaled failure tensile force for child (N)	AF cross-sectional area of child (mm^2^)	Failure stress for child (MPa)	Reference for failure tensile force
C4–C5	571	233.41	14.66	15.92	Yoganandan^64^
	1280	560.10		35.70	Kasra^26^
C6–C7	505	206.43	17.74	11.63	Yoganandan^64^
	1280	560.10		29.49	Kasra^26^

1 $$ f^{\prime} = F^{\prime}_{\hbox{max} } *\alpha^{\prime}*\alpha^{\prime}_{A} $$

where $F^{\prime}_{\hbox{max} }$ is the failure tensile force of intervertebral disc for adult, *α*′ is the scale factor for material property, $\alpha^{\prime}_{A}$ is the cross-sectional scale factor that is defined as the square of the dimensional scale factor G_S_. As listed in Table 1, *α*′ selected was 0.782 while $\alpha^{\prime}_{A}$ was 0.723. The failure forces reported by Yoganandan *et al*.^64^ and Kasra *et al*.^26^ were used to determine the corresponding failure stress (Table 2). As can be seen in Table 2, the failure stress calculated from the Kasra study was more than twice that calculated from the Yoganandan study. In order to determine a proper failure stress value, a reverse engineering approach was conducted by comparing simulation results to experimental data. More descriptions of this approach were provided in the Model validation section.

**Figure 2:**
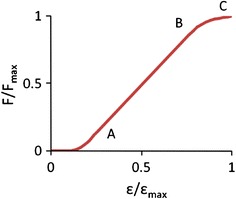
A typical force–deflection curve used to define the material property of ligaments. The curve was normalized by the failure tensile force and deflection. C is the tolerance point, A and B define a linear region in the force–deflection curve

The ligaments were modeled using tension-only bar elements. The load-deformation curves of the ligaments had a sigmoidal shape characterized by three points as shown in Fig. 2.^3^,^48^ The strain and force of the controlling points for each ligament were normalized by the failure strain and force respectively (Table 3). It is assumed that only the failure deflection and force were lower for child while the shape of the force–deflection curve was retained. The failure strain and force for adult in C2-T1 segments were provided by Yoganandan *et al*.^60^ as shown in Table 3. Yoganandan *et al*.^61^ also provided the failure deflection and force for adult in C0 (head/occipital)-C2 segments as shown in Table 4. The normalized controlling points for the C0–C2 ligaments used the data in Table 3 and the corresponding ligaments were also shown in Table 4. These failure values were used to obtain the force–deflection curves for child.

**Table 3:** Coefficients used to define the force–deflection curve for ligaments^3^ and the failure forces and strains for adult ligaments^60^

	Point A		Point B		Point C			
					C2–C5		C6–C7	
	*ε*_1_ */ε*_max_ (%)	*F*_1_ */F*_max_ (%)	*ε*_2_ */ε*_max_ (%)	*F*_2_ */F*_max_ (%)	*ε*_max_	*F*_max_ (N)	*ε*_max_	*F*_max_ (N)
ALL	21.1	10.8	77.2	85.9	0.31	93	0.35	145
PLL	25.0	9.8	77.3	77.9	0.18	71	0.34	188
CL	26.0	15.0	76.0	88.0	1.48	120	1.16	181
LF	28.6	20.9	76.2	89.3	0.77	121	0.88	129
ISL	30.8	20.1	74.4	90.9	0.61	39	0.68	39

*ε*_1_, strain at point A; *ε*_2_, strain at point B; *ε*_max_, failure tensile strain; *F*_1_, tensile force at point A; *F*_2_, tensile force at point B; *F*_max_, failure tensile force; ALL, anterior longitudinal ligament; PLL, posterior longitudinal ligament; CL, capsular ligament; LF, ligamentum flavum; ISL, interspinous ligament

**Table 4:** Failure data for ligaments in C0 to C2 of adult^43^^,^ 61_ENREF_51

Spinal level	Type	*F*_max_ (N)	*d*_max_ (mm)	F-d laws	*ε*_max_
OC–C1	JC	320	9.9	CL	2.54
OC–C1	AA-OM	232	18.9	ALL	0.68
OC–C1	PA-OM	83	18.1	LF	1.28
C1–C2	ALL	263	11.8	ALL	0.68
C1–C2	JC	314	9.3	CL	2.11
C1–C2	LF	111	9.6	LF	0.91
OC–C2	TM	76	11.9	PLL	0.41
OC–C2	Apical	214	8	ISL	0.36
OC–C2	Alar	357	14.1	ISL	2.20
OC–C2	CLV	436	12.5	CL	1.60

*F*_max_, failure tensile force; *d*_max_, failure tensile deflection; F-d laws, the corresponding ligaments used to obtain the nomalized controlling points; JC, joint capsules; AA-OM, anterior atlanto-occipital membrane; PA-OM, posterior atlanto-occipital membrane; ALL, anterior longitudinal ligament; LF, ligamentum-flavum; TM, tectorial membrane; CLV, cruciate ligament, vertical portion; PLL, posterior longitudinal ligament; CL, capsular ligament; LF, ligamentum flavum; ISL, interspinous ligament

Since ligaments of child have different dimensions and properties, the following formulation was used to calculate the three controlling points of the force–deflection curve for child ligaments. The data from Tables 3 and 4 were used in these calculations.

2 $$ \left\{ {\begin{array}{l} {d_{i} = \varepsilon_{\hbox{max} } \left( {\frac{{\varepsilon_{i} }}{{\varepsilon_{\hbox{max} } }}} \right) \times l} \\ {f_{i} = \frac{{F_{\hbox{max} } \times \left( {\frac{{F_{i} }}{{F_{\hbox{max} } }}} \right) \times \alpha_{i} \times \lambda_{iA} }}{{N_{i} }}} \\ \end{array} } \right. \quad i = 1,2,3 $$

$$ \frac{{\varepsilon_{3} }}{{\varepsilon_{\hbox{max} } }} = 1;\quad \frac{{F_{3} }}{{F_{\hbox{max} } }} = 1 $$

where *d*_*i*_ is the deflection for child ligament, *l* is the length of the ligaments in the child model, *f*_*i*_ is the force for child ligament, *α*_*i*_ is the scale factor of material property for ligaments which was 0.893 (Table 1), *λ*_*iA*_ is the scale factor of the cross-sectional area that is defined as the square of the dimensional geometrical scale factor G_S_ which was 0.723 (Table 1), *N*_*i*_ is the number of bar elements for each ligament in the child model, ε_1_ is the strain at point A, ε_2_ is the strain at point B, ε_max_ is the failure tensile strain, *F*_1_ is the tensile force at point A, *F*_2_ is the tensile force at point B, *F*_max_ is the failure tensile force.

For ligaments in the upper cervical spine, experimentally measured lengths were not provided by Yoganandan *et al*.^61^ The lengths of upper ligaments reported by Panzer^43^ in their FE model were used to calculate the failure strains for adult as shown in Table 4. The failure of ligament was simulated by deleting the corresponding element when the failure displacement reached a preset value.

The thickness of the facet cartilages for child was assumed to be 0.35 mm based on the thicknesses of facet cartilages for adult as reported by Yoganandan *et al*.^59^ The facet cartilage was modeled using hexahedral elements with an isotropic-elastic material model. The facet joint was treated as a contact problem using the surface-to-surface contact algorithm with an assumed friction coefficient of 0.1.^50^ Capsular ligaments were modeled using tension only bar elements connecting the superior aspect of the facet joint to the inferior section.

**Figure 3:**
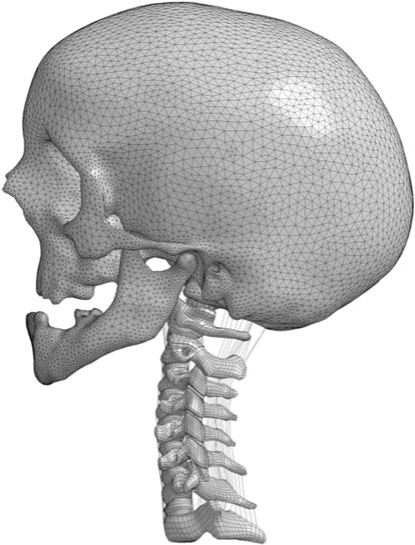
The 10-year-old ligamentous cervical spine FE model with a rigid head and T1

The head and T1 were modeled using shell and solid elements respectively and assumed to be rigid. The final ligamentous cervical spine model is shown in Fig. 3. In total, 634 1D bar elements, 20,644 2D quad shell elements, and 27,438 hexahedral elements were used to construct the entire model. The mesh quality of this child cervical spine model is shown in Table 5.

**Table 5:** Mesh quality of the 10-year-old child cervical spine model

	Jacobian		Warpage		Skew	
Solid element	≥0.5	Minimum	≤40	Maximum	≤60	Maximum
	99%	0.38	99%	49.9	100%	59.9
Shell element	≥0.7	Minimum	≤30	Maximum	≤45	Maximum
	99%	0.51	98%	49.9	99%	55.8

	Aspect ratio		Quad faces minimum angle		Quad faces maximum angle	
Solid element	≤5	Maximum	≥30	Minimum	≤150	Maximum
	99%	8.2	100%	26.6	100%	158.1
Shell element	≤3	Maximum	≥45	Minimum	≤135	Maximum
	100%	3.8	97%	30.3	97%	149.8

### Model Validation

**Figure 4:**
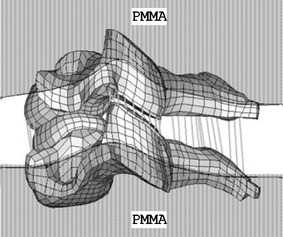
A schematic diagram of constrained area for the sub-model, similar to that conducted in tests using polymethylmethacrylate (PMMA) (C4–C5). The inferior edge was fixed and the superior edge was loaded

There was very few experimental data for child cervical spine published in the literature. Luck *et al*.^31^,^32^ and Luck^30^ conducted a series of experiments on pediatric cervical spines, with ages ranging from 20 weeks gestational to 18 years, in tension and flexion/extension. Three segments (C0–C2, C4–C5, and C6–C7) were tested in tension to determine the axial stiffness as well as displacements and forces at failure.^31^,^32^ Bending moments and angles were determined under undamaged load for the three segments in flexion and extension tests.^30^ Data from two specimens, aged nine and 12 years old, were selected to validate the ligamentous cervical spine model because of their close resemblance in age to the 10 years model. Portions of the cervical spine model were dissected to form the segmental sub-models and loaded by prescribed motion the same as those in tensile failure and bending load experiments. For the C4–C5 and C6–C7 segments, the sub-models were fixed at the inferior edge while loaded at the superior edge. A cross-sectional plane was set up near the superior edge to calculate the reaction force for tension or moment for flexion/extension, similar to that used in experiments. The constrained areas for the C4–C5 and C6–C7 segments were illustrated in Fig. 4 according to the constrained method used in the tests^31^ and communications with Dr. Luck. For C0–C2 segment simulation, the nodes that connected to the occipital bone were constrained in all degrees-of-freedom. This sub-model was loaded at the inferior edge and the cross-sectional plane was set up near the inferior edge. The area constrained for prescribed motion of C2 was the same as that for used for C5 as shown in Fig. 4. Simulations were conducted using LS-DYNA version 971 (LSTC, Livermore, CA).

Segmental tensile study reported that the failure displacement normalized by the spinal length decreased as the age increasing from birth to young adult.^32^ It was also indicated that pediatric spinal ligaments could withstand significant stretching without tearing.^34^ However, the values of failure strain were not directly reported in the literature. The stresses of intervertebral disc at failure also needed to be identified. Design of Computer Experiments (DOCE) has been used to study the effect of contributing factors.^33^ DOCE was used in the present study to analyze the effect of assumed ligament failure strain and intervertebral disc failure stress to the ultimate displacement and force in tension. To set up DOCE simulations, four levels of increased strain were selected (baseline, +25, +50 and +75%) based on published studies.^32^,^36^ Based on the calculated failure stresses for intervertebral discs listed in Table 2, seven levels of failure stresses, ranging from 15 to 45 MPa, were used. Four failure strain and seven failure stress levels were assumed for the C4–C5 and C6–C7 segments. For a full factorial analysis, these selections constitute 56 simulations. Additionally, four levels of ligamentous failure strain were assumed for the C0–C2 segment. Altogether, a total of 60 runs were simulated in tension and the ultimate displacements and forces, identified from force–displacement curves, were used for DOCE analysis. The experimentally obtained ultimate displacement and force for a 10-year-old child were calculated by interpolating the experimental values between 9-year-old and 12-year-old children.^32^ The calculated ultimate displacements were 14.4, 5.0 and 6.4 mm for C0–C2, C4–C5 and C6–C7 segments respectively. The calculated ultimate forces were 2.185, 1.277 and 1.623 kN for C0–C2, C4–C5 and C6–C7 segments respectively. Minitab (Ver, 15.0, State College, PA) was used to perform DOCE analysis. Pareto and main effect charts were used to analyze the effects of these two factors.^33^ In the Pareto chart, the horizontal bar shows the ranking of effects for each parameter and coupling. The main effect chart depicts the effects of each individual variable.

The mechanical test data for adults reported by Nightingale *et al*.^39^ were used to calculate the moment–angle’s corridors for the child. The angles and bending moments were scaled using the method reported by Irwin and Mertz.^22^ The ratio of the bending moment is equal to the cube of x direction scale factor of the neck:

3 $$ R_{\text{M}} = \lambda_{x}^{3} $$

Here the scale factor *λ*_*x*_ is 0.723, so the ratio of the bending moment is 0.38. The ratio of the angle is equal to the z direction scale factor divided by x direction scale factor:

4 $$ R_{\theta } = \frac{{\lambda_{z} }}{{\lambda_{x} }} $$

Here *λ*_*z*_ is 0.793, so this ratio of angle is 1.1.

To evaluate the response of the whole model in tension and flexion/extension, experimental data reported by Ouyang *et al*.^40^ in the testing of a 12 years old specimen were compared to simulations. In Ouyang’s tests, pure moments of ± 2.4 Nm were applied in extension and flexion and then destructive tests were conducted in tension. The data from the specimen of 12 years old was selected because of its close resemblance in age to the 10 years model. The centre of gravity (CG) of the head for the child model was determined based on that of adult provided by Walker *et al*.^52^ For the simulation of tensile test, the CG of head were constrained in the same manner as that used in the test and loaded by a prescribed motion along the z-translational degree-of-freedom. All degrees-of-freedom of T1 were constrained. For simulating the flexion and extension, the head was constrained and T1 was loaded by the prescribed motion at y-rotational degree-of-freedom. The method to determine the force or moment in C0–C2 segment was used to calculate the reaction force for tension or moment for bending. Luck^30^ calculated the bending angles of the whole cervical spine at moments of ± 0.1 Nm based on results of their three segmental flexion/extension tests. Data from the specimens of 9 years old and 12 years old were used to evaluate the child FE model responses.

## Results

**Figure 5:**
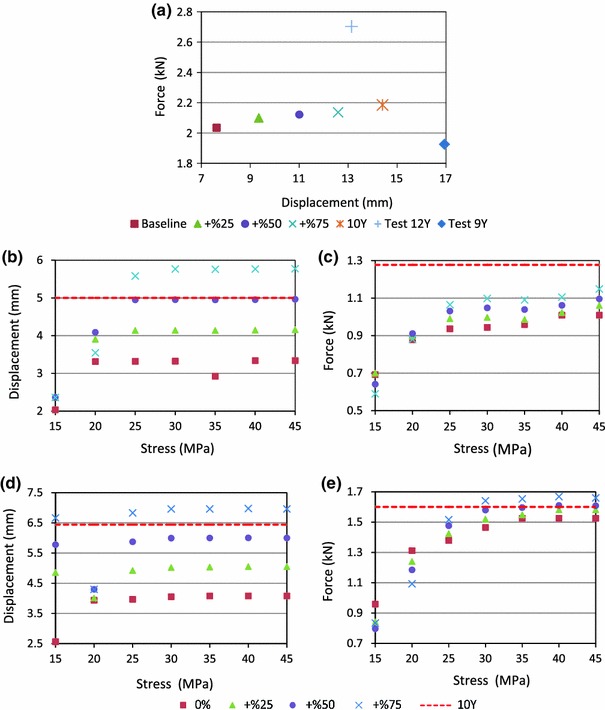
Model predicted ultimate force–displacement relationship for C0–C2 (a), ultimate displacement (b) and force (c) for C4–C5, and ultimate displacement (d) and force (e) for C6–C7. The abscissa in (b) to (e) represents the disc failure stress and the ordinate represents the ultimate displacement (b, d) or ultimate force (c, e) respectively. Baseline (0%), +%25, +%50 and +%75 were the increased percentages of assumed disc failure strain. The horizontal dashed lines represent the experimentally reported ultimate displacement in (b, d) or ultimate force (c, e) based on linear interpolation of data reported by Luck *et al*. (2013) for the 9 and 12 years old

The DOCE simulated ultimate displacements and forces were shown in Fig. 5. For the C0–C2 segment, the ultimate displacements and forces in tension increased as the ligaments failure strain increased (Fig. 5a). The simulated ultimate forces were within that obtained for the nine- and 12-year-old tests and smaller than that of calculated 10-year-old data, while all simulated ultimate displacements were smaller than those obtained from tests. The tensile stiffness was calculated using linear regression of the force–displacement curve between 10 and 90% of the failure force. The calculated stiffness values for the four runs (baseline, +25, +50 and +75%) were 324, 268, 218 and 200 N/mm. Because the measured stiffness of the 9-year-old test was 219 N/mm, which was only 0.4% higher in stiffness than that predicted by the model with an increase of 50% in failure strain, it was deemed that an increase of 50% for the failure strain of C0–C2 segment was appropriate.

**Figure 6:**
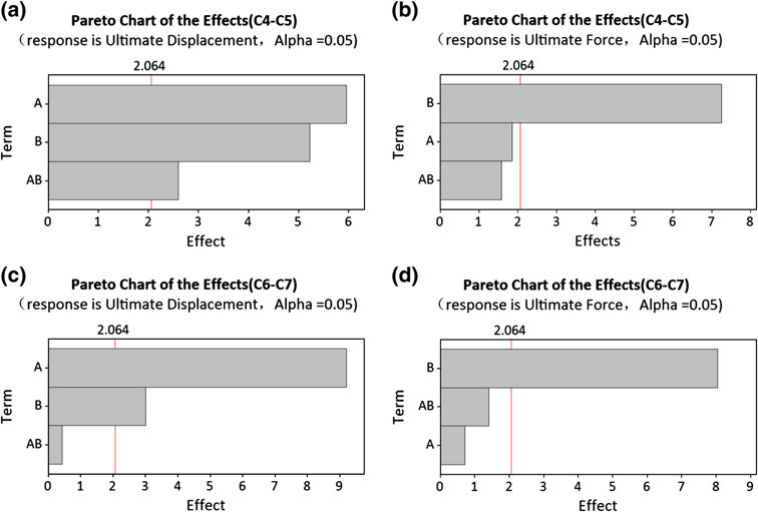
Pareto analysis for C4–C5 and C6–C7 segments (A: percentage increased for ligament failure strain, B: failure stress for intervertebral disc, and AB: combined effects)

**Figure 7:**
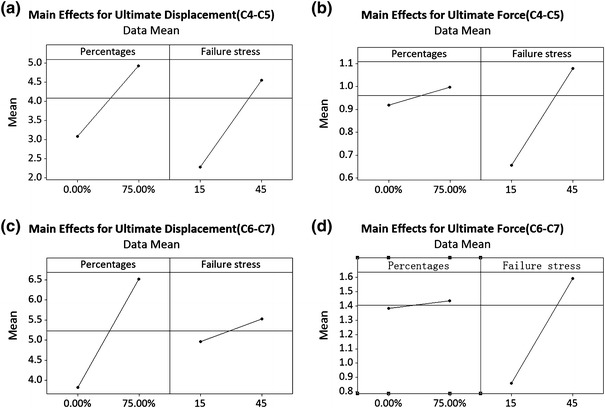
Main effects of ultimate displacement-force of tension curves for C4–C5 and C6–C7 segments

**Figure 8:**
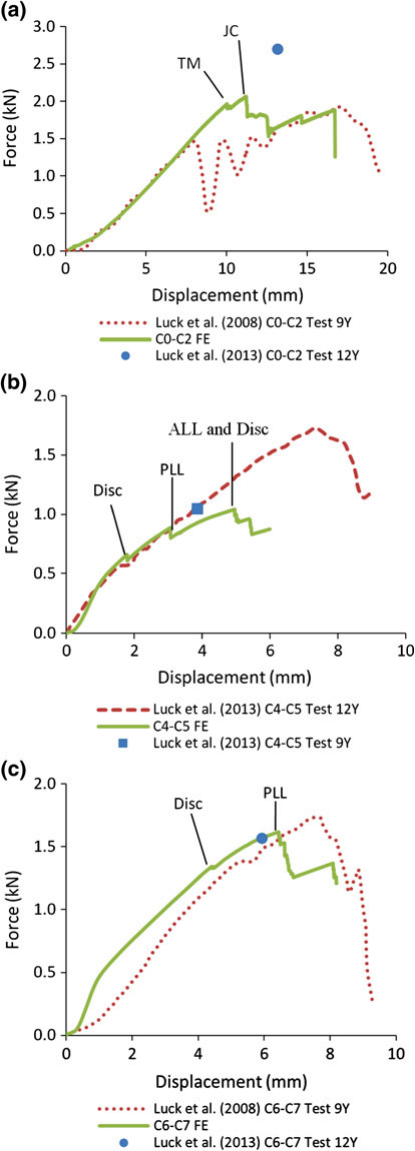
Tension response of predicted by the models vs. experimental data for the 9 years old and twelve years old at (a) C0–C2 segment, (b) C4–C5 segment, and (c) C6–C7 segment. The initial failures occurred at tectorial membrane (TM) and joint capsules (JC) ligament for C0–C2 segment, and disc, PLL and ALL for C4–C5 segment, and disc and PLL for C6–C7 segment

For the C4–C5 segment, Pareto analysis (Figs. 6a, 6b) indicated that the pre-set ligament failure strain and intervertebral disc failure stress both affected the ultimate displacements significantly and only the failure stress affected the ultimate force in a statistically significant manner. Increasing the ligaments failure strain percentage from baseline (0%) to +75% only increased the ultimate force by 0.1 kN (Fig. 7b). The disc failure stress could be determined based on the ultimate force and then the ligament failure strain could be determined based on the ultimate displacement. As shown in Fig. 5c, when the disc failure stress was assumed to be 30, 40 or 45 MPa with an assumed ligament failure strain of +50 or +75% increase, the ultimate force was close to that obtained experimentally. Considering that the calculated disc failure stress (Table 2) was between 29 and 36 MPa based on test results reported by Kasra *et al*.,^26^ a value of 30 MPa was selected. As shown in Fig. 5b, when the ligament failure strain was set at +50% and disc failure stress at 30 MPa, the ultimate displacement was close to that reported experimentally. Consequently, the failure strain for the ligament was assumed to be +50%. For the C6–C7 segment, the same relationship between the disc failure stress and ligament failure strain was observed (Figs. 6c, 6d and Figs. 7c, 7d). As shown in Figs. 5d and 5e, when the disc failure stress was larger than 30 MPa and the ligament failure strain was set at +50% level, the model-predicted ultimate force and displacement were close to experimental data. Increasing the disc failure stress from 30 to 45 MPa resulted in near identical tensile ultimate forces and displacements. Considering that the calculated disc failure stress for C6–C7 segment (Table 2) was 29.48 MPa based on test reported by Kasra *et al*.,^26^ 30 MPa was selected. For the other ligaments and discs, the ligament failure strain was also set at +50% and disc failure stress at 30 MPa. Figure 8 shows the simulated tensile force–displacement curves using these pre-determined disc failure stress and ligament failure strain.

As shown in Fig. 8a, the simulation curve had agreement with experimental curve before the first peak appeared. The force at the first peak in simulation was 25.4% higher than that of the 9-year-old obtained experimentally and the simulated ultimate force was also larger by 6.6%. However, this value was still lower than the ultimate force of the 12-year-old test. The simulated ultimate displacement was 16.4% lower than that of 12-year-old test. The tectorial membrane (TM) failed initially and the ultimate force appeared when the ligament of joint capsules (JC) failed.

For the C4–C5 segment, the simulation curve was consistent with the experimental curve before the first peak appeared (Fig. 8b). The ultimate force was 1.06 kN that was very close to 1.05 kN experimentally obtained in the 9-year-old test. The simulated ultimate displacement was 5.0 mm, which was 23.0% larger than that reported for the 9-year-old, but the displacement was still within the experimental corridor. The intervertebral disc failed partially first followed by ligament failures starting with PLL. The ultimate force appeared when ALL failed. ALL, PLL and LF bore similar forces when ligaments failure initially occurred. The disc failed at the superior growth plate and endplate cartilage of C5.

For the C6–C7 segment, the simulated ultimate force was 3.0% larger than that reported for the 12-year-old and 7.0% lower than that for the 9-year-old test (Fig. 8c). The simulated ultimate displacement was 7.9% larger than that of the 12-year-old test and 16.0% smaller than that of the 9-year-old. The intervertebral disc failed partially first followed by ligaments starting with PLL. The ultimate force appeared when PLL failed. ALL bore the largest force when ligament failure initially occurred. The disc failed at the inferior growth plate and endplate cartilage of C6.

**Figure 9:**
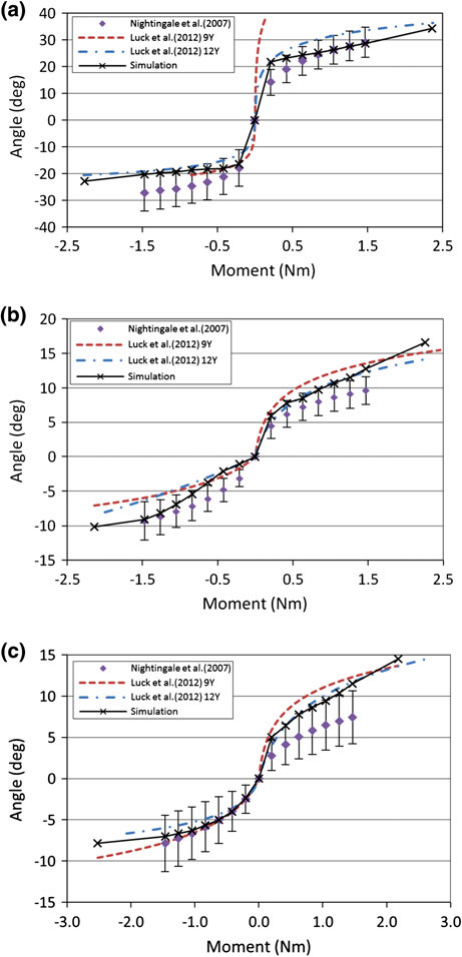
Flexion (positive) and extension (negative) response of simulation vs. children experimental responses and scaled experiment corridors at (a) C0–C2, (b) C4–C5 and (c) C6–C7

Figure 9 shows the flexion and extension simulation results compared with the children experimental data for the 9 years old and 12 years old, and the scaled experimental corridors. The simulation curves were consistent with the children experimental curves except the experimental curve of the 9 years old in flexion at the C0–C2 segment. The model was slightly more flexible compared to the child experimental response in extension at the C4–C5 segment, but the simulation curve was mostly within the scaled experimental corridor.

**Figure 10:**
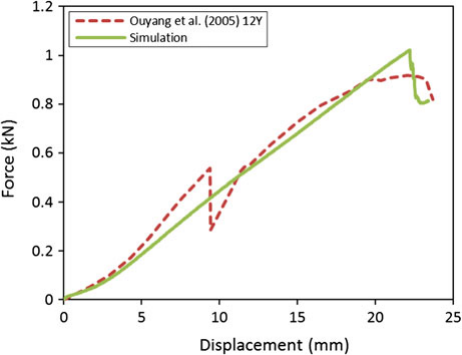
Tensile force–displacement response predicted for the whole cervical spine FE model compared to experimental data. Failures predicted by the model initially occurred at the inferior growth plate and endplate cartilage of C2 at the time that the ultimate force appeared

The model-predicted force–displacement curve for the whole cervical spine model compared with the experimental data is shown in Fig. 10. The simulated ultimate force was 10.3% larger than that of the child test. The model-predicted ultimate displacement was almost the same as that reported in the test at the time that the ultimate force occurred. However, the model did not predict the drop in force at the displacement of 9.4 mm. Additionally, the failure occurred at the inferior end-plate of C7 for the test while the simulation predicted failures at the inferior growth plate and endplate cartilage of C2.

**Figure 11:**
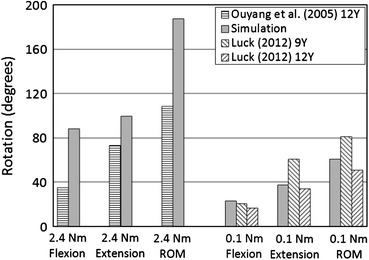
Model-predicted whole cervical spine responses to flexion and extension compared with experimental data

The model-predicted maximum rotation angles in flexion and extension for the whole cervical spine are shown in Fig. 11. Angles calculated from simulations were 152%, 35% and 73% larger than those reported by Ouyang *et al*.^40^ in flexion, extension and range of motion (ROM) respectively. Simulated results were within the range of Luck’s data except that the maximum flexion angle was 13% larger than the experimental data of 9 years old.

## Discussion and Conclusions

A 10-year-old child ligamentous cervical spine FE model was developed using detailed geometry of a pediatric subject and material properties derived specifically for a ten-yer-old subject. The material properties were mostly obtained by scaling down the adult experimental data using the scale factors based on literature. The model was validated in tension and further exercised in flexion and extension against human cadaveric data.^30^–^32^,^40^ The failure response was validated in tension based on experimental results.^31^,^32^

The failure displacement for each ligament was assumed for all bar elements representing the ligament. The percentage increased in strain was determined using the DOCE analysis method. Dibb *et al*.^10^ reported that the ultimate displacements for adults were 10.8 ± 3.9, 7.7 ± 2, and 7.8 ± 1.7 mm for C0–C2, C4–C5 and C6–C7 segments, respectively. The ultimate displacements for the 9-year-old and 12-year-old child reported by Luck *et al*.^32^ were within this deviation of adult results. In the current study, the failure displacements of ligaments did not exceed the values of the adult after an increase of 50% for the failure strain.

For failure simulation of the intervertebral disc, the element failure method was used by defining critical stresses for the materials of growth plate and endplate cartilage. DeWit and Cronin^9^ used a contact-break method to simulate disc failure by defining the failure stresses based on the study of Kasra *et al*.^26^ The tension failure force and displacement fell outside the corridors reported by Dibb*et al*.^10^ It was assumed that the contact-failure method caused the distinct results. The simulated ultimate forces and displacements for the current child model were consistent with experimental data. It may reveal that the element failure method was better than the contact-break method to define disc failure. The failure stress for the C4–C5 and C6–C7 segments was 30 MPa, which was close to the calculated failure stresses based on experimental data provided by Kasra *et al*.^26^

The DOCE results indicated that increasing the failure stress of the intervertebral disc did not increase the ultimate displacement or force when the failure stress increased to a certain value (Fig. 5). The reason may be that the peak of the tension curves was controlled by ligaments. When a certain ligament failed, even though the intervertebral disc was not ruptured, a peak appeared in the tension curve. This can be proven by the tension results in Figs. 8b and 8c. All the peaks of the curves appeared when ALL or PLL failed.

The model-predicted tensile force–displacement curves were compared with experimental data using the pre-determined failure strain for ligament and stress for disc (Fig. 8). The failure initially occurred at the intervertebral disc, followed by PLL and ALL for the C4–C5 and C6–C7 segments. The same prediction was found in the study of DeWit and Cronin^9^ who validated their adult FE C4–C5 segment model in tension. The injury descriptions for the neck segment due to tensile loading were provided by Luck.^30^ Fractures of the dens and left condyle occurred for the C0–C2 segment; For the C4–C5 segment, the physis endplate failure occurred at C4 or C5; For the C6–C7 segment, the physis endplate failure occurred at C6. Similar failure locations in intervertebral discs for the C4–C5 and C6–C7 segments were found in the failure simulations. For the C0–C2 segment, the major failure force (first peak) was higher in the tensile simulation curve compared to experimental data (Fig. 8a). The reason may be that bone failure was not considered in the current model but it was apparent in tests. The failure of bone (such as dens) might change the tensile force distribution to the ligaments.

The whole FE model was simulated in tension and compared to experimental data reported by Ouyang *et al*.^40^ The model applied the material properties determined in segmental validation processes without additional changes. The model-predicted force–displacement curve was consistent with the experimental data. This indicates that these material properties are acceptable for use in tension and the simulation is able to predict the tension force and displacement. The reason that the model did not predict a drop in force at a displacement of 9.4 mm may be related to the partial failure of soft tissue at smaller displacement failed to capture in the simulation.

The method provided by Irwin and Mertz^22^was used to obtain the experiment corridors for child in flexion and extension. Irwin and Mertz^22^ developed the method to scale down the adult experimental data for child dummy validation. This method considered the differences in geometry, mass, and material properties between child and adult.^35^ Mertz *et al*.^35^ obtained the response corridors for the Hybrid III 10-year-old dummy by scaling adult corridors using this method. The dummies’ neck response was acceptable in flexion and extension. Meyer *et al*.^36^ used this method to scale the volunteer response corridors in frontal, oblique, and lateral impacts. In current study, the children experimental data reported by Luck^30^ were almost within these scaled corridors but were mostly close to the edges of the corridors (Fig. 9). The reason may be that the initial positions of the tested segments were different between the children test and the adult test reported by Nightingale *et al*.^39^ or the limitation of scaling method.

The whole FE cervical spine model behaved similar to those reported by Luck^30^ in flexion and extension, but was much larger than the data published by Ouyang *et al*.^40^ Luck indicated that angles they calculated were much larger than those reported by Ouyang *et al*. for the older child specimens (6–12 years). The same results were observed from the FE model simulation. The predicted ROM was 73% larger than the data reported by Ouyang *et al*.

As most of human models, limitations exist in this study. The primary ones are related to material properties and experimental data. Since there were no appropriate cadaveric material property data for the child, the scaling method was used to assume the material property data for the child model. The accuracy of the scale factors were also limited by the lack of material data. The current model could not be used to simulate the experimentally observed gradual failure of ligaments. The experimental data used in tension and flexion/extension validation came from only two specimens for three segments and one specimen for whole ligamentous cervical spine. Since no experimental data exists for validation, the bone failure was not considered in the current study.

Only few FE child cervical spine models have been developed, partially because of lack of experimental data. Based on limited available child experimental data, a ten YO child ligamentous cervical spine FE model was developed and validated. Advanced material laws were used to define the material properties which were all based on existing data. This model provided a good prediction of responses over tension, flexion, and extension. The failure properties were validated in tensile loading based on published experimental data. It should be noted that only tension and flexion/extension loading conditions were simulated in the current study. More development and validation works still need to be carried out before this model can be integrated into a whole body child model in order to predict child neck injuries in car crashes and improve the vehicle design. Future studies will include evaluation of responses at the segment level in more loading scenarios and modeling of the full cervical spine with muscles for crash injury analysis.
